# Evolution of the occurrence of *Tityus serrulatus* (LUTZ & MELLO, 1992) in the state of Santa Catarina

**DOI:** 10.1590/0037-8682-0434-2022

**Published:** 2023-02-20

**Authors:** Taciana Mara da Silva Seemann, Ana Paula da Rocha, Fabíola Cremonese, Marisete Canello Resener, Andrea Petry, Ana Carolina Conchon Costa

**Affiliations:** 1 Universidade Federal de Santa Catarina, Secretaria de Estado da Saúde de Santa Catarina, Centro de Informação e Assistência Toxicológica de Santa Catarina, Florianópolis, SC, Brasil.; 2 Universidade Federal de Santa Catarina, Departamento de Ciências Farmacêuticas, Florianópolis, SC, Brasil.; 3 Universidade Federal de Santa Catarina, Hospital Universitário Polydoro Ernani de São Thiago, Unidade de Terapia semi-intensiva e Intensiva Neonatal, Florianópolis, SC, Brasil.

**Keywords:** Tityus serrulatus, Scorpionism, Epidemiology, Endemic

## Abstract

**Background:**

Scorpions are a leading cause of envenomation in Brazil. The species *Tityus serrulatus* is associated with the most severe cases, especially in children. Despite not being endemic to the state of Santa Catarina, such occurrences have increased more than 500% in the state recently. Therefore, this study aimed to analyze the occurrence of envenomation by *T. serrulatus*, attended by the Center for Toxicological Information and Assistance of Santa Catarina.

**Methods:**

This was a retrospective and descriptive study of the occurrence of *T. serrulatus*, identified by the agency, from 2014 to 2021 in Santa Catarina, using data obtained by the BI-DATATOX system.

**Results:**

A total of 112 occurrences were classified as envenomation. Of these cases, 48.2% were recorded in the Itajaí Valley region and 33% in Greater Florianópolis. Men were involved in 59.8% of these, and the most common age group was 20-39 years (39.3%). Most envenomation occurred in urban areas (89.3%) under non-occupational circumstances (83%). Stings were more frequent on the hands (50.9%). Care was sought within 1 h after the event in 75.9% of the cases, and 94.6% were classified as mild.

**Conclusions:**

Occurrence of envenomation involving *T. serrulatus* in Santa Catarina increased significantly during the study period. Most cases occurred in urbanized areas, which suggests that they might have been transported from other states, and it must be considered that, in the urban environment, scorpions find a large supply of food and shelter and a reduced number of specific predators, allied to parthenogenesis.

## INTRODUCTION

**FIGURE 1:**
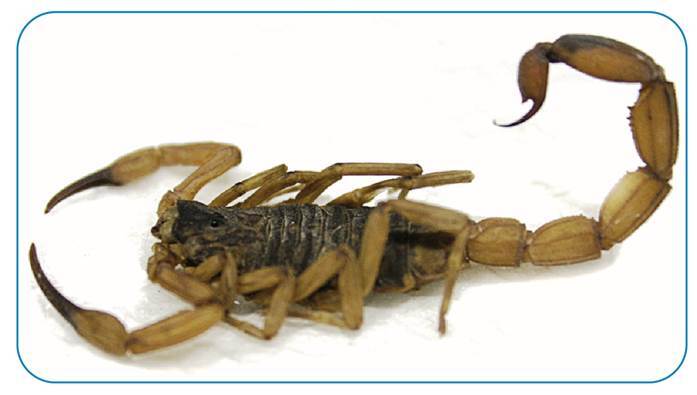
*Tityus serrulatus* or “yellow scorpion” **Legend:** Image from CIATox/SC.

In Brazil, approximately 180 species of scorpions are found; they belong to the families Bothriuridae, Chactidae, Hormuridae, and Buthidae. The latter houses the genus *Tityus*, including the species of greatest clinical importance in Brazil, *Tityus serrulatus*; its main morphological characteristics are serrate-forming granules in the third and fourth segments of the metasome^1^. The species, also known as the yellow scorpion, has nocturnal habits and moves in normally dry and sandy environments. Their length vary from 6 to 7 cm. Animals present a light-yellow color, with dark trunks and carapace, without spots on the legs and pedipalps^2^ (Figure 1).

Envenomation with scorpions occupy the first position among the data of serious accidents with venomous animals, representing more than 50% of the injuries of this type, according to statistics from the Sistema Nacional de Informação de Agravos (National Disease Notification System) - SINAN^3^, and increased by 576% in Santa Catarina in the last 20 years, according to data from the Brazilian Ministry of Health^4^. Clinical warning regarding scorpionism concerns the pathophysiological and multisystemic effects that result from the sting. The venom of scorpions of the genus *Tityus* is composed of hyaluronidase proteins, amino acids, and salts, which can cause toxic effects in patients. The action on sodium channels results in tissue neurotoxic activity causing depolarization of membranes and, consequently, the release of neurotransmitters, producing adrenergic and cholinergic effects in various organs with varied intensity. Cardiovascular effects such as hypo-and hypertension, arrhythmias, direct cardiotoxicity, and acute lung edema are described as symptoms resulting from scorpion stings. Respiratory, neuromuscular, and gastrointestinal changes such as excessive salivation and increased intestinal motility have also been reported. Hematological responses such as platelet aggregation and inhibition of the angiotensin enzyme, as well as metabolic signs such as hyperglycemia and hydroelectrolytic disorders, can also be included in addition to effects on the central nervous system, such as shock and systemic and cerebral hypoxia^5^.

In addition to the pathophysiology of scorpion stings, there is a concern at the public health level, mainly because of how the yellow scorpion reproduces. *T. serrulatus* is a parthenogenetic and ecologically opportunist; thus, it can easily adapt to new environmental conditions^1^. A single female can have an average of two births per year, with an average of 18-25 young scorpions per gestation, which makes control work difficult. The species originally lived in transition forest, dry forest, savanna, and caatinga environments, a restricted area in the state of Minas Gerais^6^ ^,^ ^7^. Currently, this species lives in habitats with minimal vegetation, being an endemic species in Brazil, and proliferates widely in states such as Bahia, Espírito Santo, Minas Gerais, Rio de Janeiro, São Paulo, Paraná, and Goiás, covering the northeast, midwest, and southeast regions of Brazil^2^. 

This spread may be due to the high potential for dispersal and colonization associated with parthenogenetic species, in addition to other causes, such as the capacity to reach human environments by roads and railways, a large reserve of populations, and the ability to feed on resources that may be abundant^8^. According to the social indicators of the Instituto Brasileiro de Geografia e Estatística (IBGE) for 2018, approximately 10% of the Brazilian population does not have access to direct or indirect garbage collection, and approximately 37% do not have access to a sanitary sewer system^9^. These conditions, associated with habitats in the urban context, normally in areas with rubble, garbage, sewage, and access to abundant feeding in the form of small animals such as spiders, cockroaches, and other insects, contribute to the dissemination of *T. serrulatus* and hinder envenomation control and prevention. These may be the reasons for the increase in the number of envenomation recorded in Brazil, which is why monitoring and controlling the species is of such importance in the field of public health.

This descriptive study aimed to comprehend and describe the dispersion behavior of *T. serrulatus* in Santa Catarina to reduce the occurrence of envenomation and encourage administrative actions, such as the implementation of control actions.

## METHODS

A study of envenoming involving the species *T. serrulatus* attended by the Center for Toxicological Information and Assistance of Santa Catarina (CIATox/SC) from 2014 to 2021, with the specimens identified by picture or received and identified in the agency, was performed using data obtained from the open-source DATATOX BI Pentaho/Saiku, version 2.6, a database from the CIATox/SC. Statistical analyses were performed using Microsoft Excel (version 2002) and open-source Epidemiologic Statistics for Public Health (OpenEpi), version 3.01. 

The DATATOX registration and monitoring system is used in the service routine of the CIATox/SC, which attends all 295 cities of Santa Catarina via a toll-free line, providing guidance in the diagnosis and treatment of poisoning and envenomation. The notification and feeding of the system are simultaneous, and the information is highly reliable both for care and for use in clinical epidemiological studies and national assessments of the impact of toxic agents on the health of the population. Data analysis can be performed from different perspectives using relational and dimensional dynamics of information, as well as through data refinement techniques that explore large volumes of data and search for correlations and facts that are not readily observable. The different variables included in this study were the region of exposure, circumstances of exposure, the severity of the cases, sex, age, sting location, and time between envenoming and care.

The notifications were grouped into annual totals by the city of exposure and then transformed into relative values. In this case, a coefficient or incidence rate was extracted, which was obtained from the ratio between the number of reported cases and the proportion per 100,000 people per city, calculated using the following formula:

$$Incidence\;rate=\frac{\left(total\;of\;reported\;cases\;\times 100,000\right)}{Total\;population\;of\;the\;city}$$

The incidence rate was also represented cartographically using choropleth maps, where the technique of natural breaks was applied to groups with similar values and maximized the differences between classes. All maps were prepared using a geographic information system in the QGis software.

## RESULTS

**FIGURE 2:**
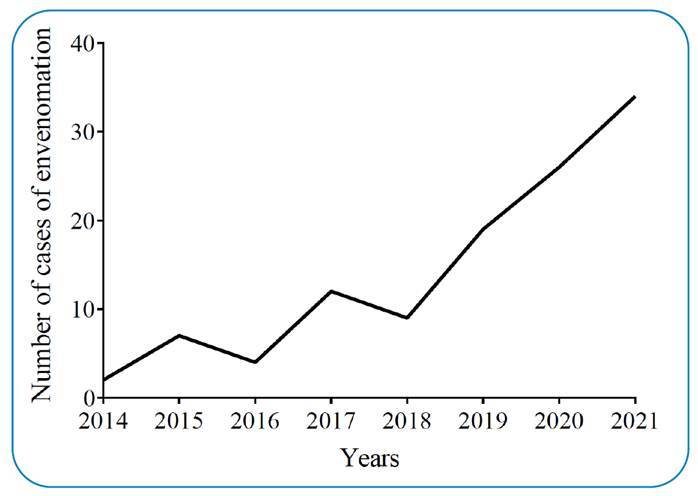
Number of envenomation with *T. serrulatu*s in Santa Catarina from 2014 to 2021. **Legend:** Data are presented as the number of cases with *T. serrulatus* from the years 2014 to 2021 in Santa Catarina (n = 112).

**FIGURE 3:**
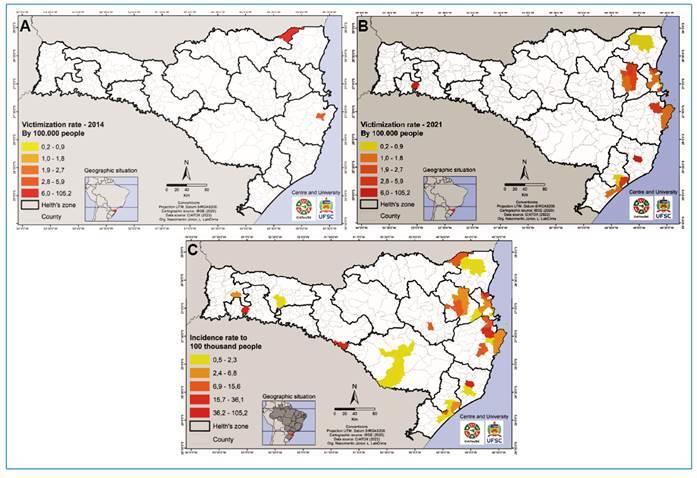
Envenomation with *T. serrulatus* in the regions of the state of Santa Catarina from 2014 to 2021. Legend: Data are presented as the incidence rate (ratio between reported cases and the proportion per 100,000 people per municipality) of the cases with *T. serrulatus* in Santa Catarina from 2014 to 2021 (n = 112). **(A)** incidence rate of envenomation with *T. serrulatus* in 2014; **(B)** incidence rate of envenomation with *T. serrulatus* in 2021; **(C)** incidence rate of envenomation with *T. serrulatus* from 2014 to 2021. **Km:** kilometer; **IBGE:** Instituto Brasileiro de Geografia e Estatística; **CIATOX:** Centro de Informações e Assistência Toxicológica; **Org:** organizer.

From 2014 to 2021, a total of 134 notifications with *T. serrulatus* were recorded by the CIATox/SC, 112 of which were envenomation and 22 were information requests in which *T. serrulatus* was identified. In 2014, only 2 cases were recorded, whereas 34 were recorded in 2021 (Figure 2). Of the 112 cases analyzed, the Itajaí Valley region comprised the largest number of envenomation involving *T. serrulatus* in the state of Santa Catarina (48.2%), followed by the Greater Florianópolis (33%), north of the state (6.3%), south of the state (5.3%), west of the state (2.7%), and middle west and the highlands regions (1.8%), and this pattern was somewhat constant during the years analyzed (Figure 3). Of the cases, 89.3% occurred in an urban area, 83% occurred in non-occupational contexts, and 17% were related to labor. Regarding envenoming in the occupational sphere, most victims were employees of maintenance and repair services (31.6%) and industrial services (15.8%). Regarding the severity of envenomation, 94.6% of cases were classified as mild, 4.5% as moderate, and 0.9% as severe. No deaths occurred during the period analyzed. Regarding the distribution of cases by sex, men were more affected than women (59.8% vs. 40.2%) (Table 1).

**TABLE 1:** Demographic data of accidents by *T. serrulatus* (2014-2021; n = 112).

Variables	n	% (95% CI)
**Region of exposure**		
Urban	100	89.3 (82.5-94)
Rural	7	6.2 (2.8-12)
Undetermined	5	4.5 (1.7-9.6)
**Circumstance of exposure**		
Non-occupational	93	83 (75.2-89.1)
Occupational	19	17 (10.8-24.8)
maintenance and repair services	6	31.6
industrial services	3	15.8
commerce (stores, markets)	2	10.5
food producers	1	5.2
transport	1	5.2
**Severity classification**		
Mild	106	94.6 (89.2-97.8)
Moderate	5	4.5 (1.6-9.6)
Severe	1	0.9 (0.04-4.3)
**Sex**		
Men	67	59.8 (50.5-68.6)
Women	45	40.2 (31.4-49.4)
**Anatomical region of sting**		
Hands	57	50.9 (41.7-60.1)
Feet	25	22.3 (15.3-30.7)
Lower limbs	17	15.2 (9.4-22.7)
Upper limbs	7	6.2 (2.8-12)
Head and abdomen	7	6.2 (2.8-12)
**Time between envenoming and care**		
< 30 min	31	27.7 (20-36.5)
31-59 min	54	48.2 (39.1-57.5)
1-5 h	18	16.1 (10.1-23.8)
> 5 h	7	6.2 (2.8-12)

Data are presented as the number of cases and percentages (95% CI). **N:** number of cases; **CI:** confidence interval; **min:** minutes; **h:** hours.

**FIGURE 4:**
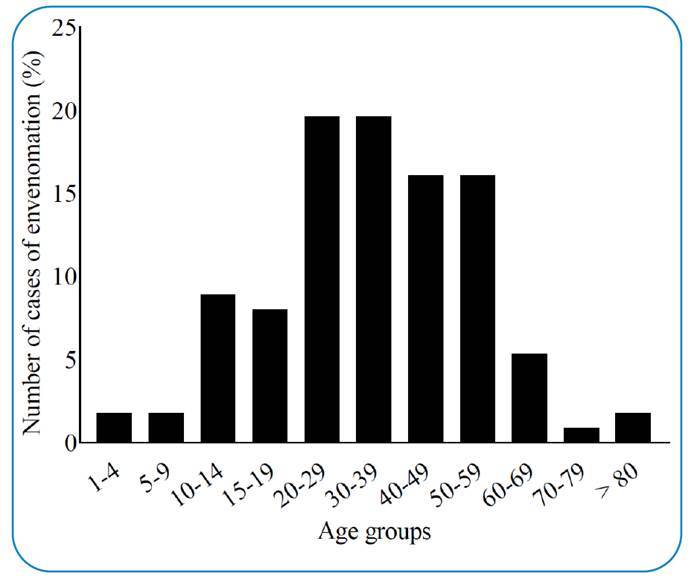
Age groups affected by scorpionism from *T. serrulatus* in the state of Santa Catarina from 2014 to 2021. **Legend:** Data are presented as percentage from the total number of cases with *T. serrulatus* in Santa Catarina from 2014 to 2021 (n = 112).

The age group 20-39 years was the most common (39.3%), followed by the age groups 40-59 (32.1%), 10-14 (8.9%), and 15-19 years (8.0%), and the least frequent groups were children aged 1-9 years (3.6%) and patients aged 70 years and older (2.7%) (Figure 4). Regarding the location of the sting, the hands were the most affected sites (50.9%), followed by the feet (22.3%), and the least affected sites were the head and abdomen (6.2%). The time elapsed between envenoming and contact with CIATox/SC and specialized care by CIATox/SC was 30 min or less in 27.7% of the cases and from 31 to 60 min in 48.2% of the cases. Only 6.2% of the victims took more than 5 h to obtain specialized care (Table 1). 

## DISCUSSION

This is the first study showing an increase in envenomation with *T. serrulatus* in the state of Santa Catarina. The majority of the cases were mild (94.6%), with 4.5% of the cases being moderate and 0.9% severe. Other studies have also reported a higher incidence of mild cases in several regions of Brazil, while severe cases varied from 0.4% to 13.5%^10^ ^-^ ^13^. Envenoming with *T. serrulatus* registered in Santa Catarina did not evolve severely, which can be associated with the lower number of cases with children in this state, since other states show a higher prevalence in children aged 0-12 years old^10^ ^-^ ^16^ and *T. serrulatus* is involved in most severe cases and fatal outcomes, especially when the victims are children^17^. This was also observed in other studies, which reported a greater volume of envenomation classified as serious among children aged 4-8 years and the occurrence of one pediatric death, which evolved in less than 24 h to acute pulmonary edema and cardiogenic shock^14^. Adolescents may also be more prone to severe cases, as shown by an analysis of variables related to the lethal evolution of scorpionism in children and adolescents from 2001 to 2005, carried out in the state of Minas Gerais, with a rate of 77% of severe cases among children aged 5-9 years and a lethality of 1.4% in children aged 1-4 years^18^. Our data showed a higher prevalence in the age groups 20-39 (39.3%) and 40-59 years (32.1%), and a lower prevalence in infants and children aged 1-9 years (3.6%) and patients aged 70 years and older (2.7%). A previous study by Silva et al. detected in their experiment a venom of *T. serrulatus* from the state of Bahia that was 7.7 times less toxic when compared with specimens from southeastern of Brazil^19^, which suggests different levels of toxicity arising from different scorpion sizes, feeding regimes, and habitats. This phenomenon may also occur in *T. serrulatus* in the south and more specifically, in Santa Catarina. However, further experimental research is required to validate this hypothesis. 

In this study, most cases took 31-60 min from the time of envenoming until the victim had medical assistance (48.2%), with the shortest time being up to 30 min (27.7%). These results are similar to those observed in other studies, which showed that most victims had specialized assistance in less than 1 h after the sting^10^ ^,^ ^14^ ^,^ ^15^. Lisboa observed a higher incidence in the time elapsed before care from 1 to 3 h after envenomation (39.3%)^13^. Comparing these data with SINAN's statistics^3^, we noticed a constant relationship between time of envenoming × time until health care. A fragment of the same period adopted for this study, extracted from SINAN at the national level, showed that in 58.2% of the cases, patients sought care between 0 and 1 h after the sting^3^. The region of Brazil with the highest notifications of this type is the southeast, with 29.6% of notifications, followed by the northeast (21.9%), midwest (3.34%), south (1.83%), and north (1.55%). For the time interval between 1 and 3 h, a reduction in the percentage (22%) could be observed, and the region that leads in this aspect is the northeast (10.7%), followed by the southeast (8.7%), midwest (1.15%), north (1.04%), and finally the south, with 0.44% of notifications between 1 and 3 h after sting^3^. Based on these data, it is not possible to state that there is a greater awareness of the importance of rapid care in scorpion envenoming in a given region compared with others, as the number of cases follows the ranking of the search for care, which makes the equation directly proportional, making such analysis difficult. 

Our data showed that the Itajaí Valley had the highest incidence of envenomation with *T. serrulatus* (48.2%), with the highest prevalence in the urban area (89.3%). This higher number of cases in urban areas seems to be a reality in the epidemiology of scorpionism in Brazil, which was also observed in studies carried out in the countryside of the states of Bahia, Alagoas, Ceará, and in the city of São José do Rio Preto. These studies showed a prevalence of 73-94.7%^10^ ^-^ ^12^ ^,^ ^14^ of the cases in urban areas, although one study performed in the south of Bahia observed a higher incidence in rural areas (62.5%) than in urban areas (32%)^13^. 

According to Porto and Brasil, most scorpion species have specific requirements for habitat^20^. On the other hand, *T. serrulatus* has high ecological plasticity, largely due to parthenogenesis, which favors the occupation of environments modified by humans^1^ ^,^ ^8^ ^,^ ^20^. Another reason for the ecological plasticity of *T. serrulatus* might be its opportunistic ecological category. Other genera are also included in this category, such as *Centruroides* and *Isometrus*. These scorpions have a short embryonic development, short lifespan, high population density, and rapid mobility. They can also multiply in single insemination and have elaborate resources for sperm storage^21^. The other points that may contribute to the occurrences in the period are intertwined in some way because they involve cargo transportation, agricultural and industrial production, territorial connections, and anthropic action^1^ ^,^ ^22^. Three important highways cross the geographical position of Santa Catarina territory: BR-282, which connects the regions of Greater Florianópolis and the West; BR-470, which links the city of Navegantes to Camaquã in the Rio Grande do Sul; and BR-101, which starts in the city of Palhoça in the metropolitan area of Florianópolis and goes north, crossing the towns of Biguaçu, Porto Belo, Camboriú, Araquari, and Joinville in the northern region of the state^23^. These roads transport the growing agricultural and industrial production in the state. Santa Catarina also has port connections with other states and countries involved in the logistics of transporting goods^24^. As mentioned previously, the species is very plastic and has been reaching an increasing number of urban and human environments in search of dry hiding places with little light and abundant food, elements that are present in trucks, boxes, and containers that travel between cities, states, and countries, and may carry scorpions and other synanthropic animals. Added to the ease of finding shelter and food, there is a lower number of specific predators in urban environments, which also contributes to the growth of yellow scorpions in the human environment. 

Envenomation was more prevalent in men than in women (59.8% vs. 40.2%), which was also reported by Lisboa and Bucaretchi^13^ ^,^ ^25^, while a higher incidence in women was observed by Carmo (54.9% vs. 45.1%) and Taniele-Silva (61.8% vs. 38.2%)^10^ ^,^ ^11^. Observing the mapping of the residential areas most prone to the occurrence of scorpions, it is clear that all inhabitants of a residence are equally susceptible to stings by scorpions, which is in accordance with our findings, considering that the differences in susceptibility between men and women are not statistically significant^1^. 

The most affected anatomical regions were the hands (50.9%) and feet (22.3%), and the least affected were the head and abdomen (6.2%). Some studies have also observed that the hands and feet were most frequently affected^13^ ^,^ ^15^. Other studies have shown a higher prevalence in the lower limbs or upper limbs^10^ ^,^ ^11^. These findings could be related to the mapping of indoor and outdoor residential areas^4^. The higher incidence of hand stings in scorpions envenoming may be linked to the wide range of suitable locations for their accommodation and reproduction, some of which are used to perform manual tasks with unprotected hands.

*T. serrulatus* is not an endemic species of Santa Catarina, and it is associated with the territorial characteristics of the state (border areas, geographic position in the context of land transport of agricultural and industrial products, and port infrastructure). These features indicate the relevance of this survey to help health surveillance gain knowledge on the distribution of this species and control diseases in the context of public health. It is important to highlight that all cases of envenomation and identification of this species attended by the CIATox/SC are communicated to the State Epidemiological Surveillance Service to assist in the program of Health Surveillance Actions in the state of Santa Catarina, which allows for the population control of *T. serrulatus* in our region. 

This study has some limitations. The number of cases obtained from our database was small, which might be a reason for the lack of severe cases. Underreporting must also be considered because the actual amount of envenomation with *T. serrulatus* may be higher than that recorded. Furthermore, although the location of exposure was the variable used in this study, we could not confirm whether the information passed to the CIATox was correct. Additionally, the venom of *T. serrulatus* from Santa Catarina was not studied to verify whether it was less toxic, which could also be a reason for most cases being mild. In addition, although not a limitation, the variables analyzed might be considered basal since this is an initial study, and these variables are an introduction to what can be explored and amplified. 

In conclusion, there was an increase in the number of cases within the period analyzed; coastal areas had higher incidences of envenomation with *T. serrulatus*, and most cases occurred in urban areas and non-occupational contexts. The most affected age group was adults aged 20-39 years. Men were more affected than women, and most cases received healthcare attention up to 1 h after envenoming. Most cases were mild, probably due to a lower incidence of envenomation in children or a less lethal variety of venom already observed in specimens in Bahia. This assumption should be better examined through experiments performed with venom obtained from yellow scorpions in Santa Catarina. 

Given the wide range of contexts and biases that permeate the occurrence and dispersion of *T. serrulatus,* we can also conclude that the identification and knowledge of the distribution of this species will help improve the management of health surveillance, zoonosis services, entomology centers, the care and information provided by the Toxicological Information and Assistance Centers, and the work of health management authorities. This allows actions to be planned, created, and sedimented with the actors who participate in them, aiming to prevent a disorderly proliferation of the species, especially near large urban centers and communities. 
